# Association of gallbladder diseases with risk of gastrointestinal polyps

**DOI:** 10.1186/s12876-022-02566-6

**Published:** 2022-11-21

**Authors:** Wenbin Geng, Xiangrong Qin, Peng Yang, Junmei Wang, Jing Yu, Xiaoyong Wang

**Affiliations:** 1 Department of Gastroenterology, The Affiliated Changzhou No. 2 People’s Hospital of Nanjing Medical University, 29 Xinglong Lane, Tianning District, Changzhou, Jiangsu Province, 213000 China; 2 Graduate school, Dalian Medical University, 9 West Section of Lvshun South Road, Lvshunkou District, Dalian, 116000 Liaoning Province China

**Keywords:** Gallbladder polyps, gastric polyps, colorectal polyps, colonoscopy, gastroscopy

## Abstract

**Background:**

It has not yet been determined whether gastroscopy and colonoscopy screening help patients with gallbladder diseases. We aim to retrospectively investigate the relationship between gallbladder diseases and gastrointestinal polyps in order to provide a theoretical basis for the early screening of gastrointestinal polyps in patients with gallbladder disease.

**Methods:**

This is a retrospective cross-sectional study involving 1662 patients who underwent gastroscopy, colonoscopy, and abdominal ultrasound as part of their health check-up from January 2015 to July 2020. We also compared the patients with and without gallbladder diseases to determine the prevalence of gastrointestinal polyps.

**Results:**

Patients with gallbladder polyps had greater odds of having colorectal polyps (adjusted odds ratio (OR)=1.77, 95% confidence interval [Cl]: 1.23 to 2.54, *p=*0.002) and gastric plus colorectal polyps (adjusted OR=2.94, 95%Cl: 1.62 to 5.32, *p<*0.001) than those without. Patients with multiple gallbladder polyps had greater odds of having colorectal polyps (adjusted OR=2.33, 95% CI: 1.33 to 4.07, *p=*0.003) and gastric plus colorectal polyps (adjusted OR=3.95, 95% CI: 1.72 to 9.11, *p=*0.001), and patients with gallbladder polyps had greater odds of having left-colon polyps (adjusted OR=1.90, 95% CI: 1.25 to 2.88, *p=*0.003) and colorectal adenoma (adjusted OR=1.78, 95% CI: 1.19 to 2.66, *p=*0.005). We also noted that women with gallbladder polyps had a higher prevalence of colorectal polyps (OR=2.13, 95% CI: 1.20 to 3.77, *p=*0.010) and gastric plus colorectal polyps (OR=3.69, 95% CI: 1.58 to 8.62, *p=*0.003). However, no positive correlation was observed between gallbladder stones and gastrointestinal polyps.

**Conclusions:**

Gallbladder polyps are significant indicators of colorectal and gastric plus colorectal polyps. Hence, gastroscopy and colonoscopy screening should be performed for patients with gallbladder polyps, particularly female patients and those with multiple gallbladder polyps.

**Supplementary Information:**

The online version contains supplementary material available at 10.1186/s12876-022-02566-6.

## Introduction

Gastric cancer and colorectal cancer (CRC) are among the most common cancers globally and are major reasons for cancer-related deaths worldwide [1]. Gastric polyps and colorectal polyps are precancerous lesions [2, 3]; hence, their early detection and removal can prevent them turning into gastric cancer and CRC. Consequently, identification of the risk factors associated with gastrointestinal polyps has increasingly attracted the attention of the research community to develop methods for preventing and screening gastrointestinal cancer.

Gallbladder disease is one of the most common conditions worldwide. We define gallbladder disease as gallstones or gallbladder polyps. Gallstone disease, a major gallbladder disease, affects 10–20% of the global adult population [4]. A study in China reported the overall prevalence of gallbladder polyps to be 6.9% in the country [5]. Gallbladder diseases are often diagnosed with abdominal ultrasound. However, in most cases, their clinical significance is unclear. Gallbladder diseases and colorectal polyps have some common risk factors such as older age, obesity, metabolic syndrome, glucose intolerance, and hyperlipidemia [4, 6, 7]. In addition, the gallbladder epithelium and the colorectal mucosal epithelium have a common epitope [8]. Consequently, the association between gallbladder diseases and colorectal polyps has aroused the interest of many clinicians. Jeun et al. suggested that gallbladder polyps may not be related to colorectal adenoma [9], but others have shown that they are indeed correlated to colorectal polyps or adenoma [10, 11]. Similarly, Liu et al. [10] and Yamaji et al. [12] revealed that gallstones are related to colorectal adenoma, whereas others [9, 13] have suggested no such correlation. A meta-analysis [14] of 24 case-control studies suggests that patients with gastric polyps show a higher occurrence of colorectal polyps. Another meta-analysis showed that symptomatic gallstones are related to gastric cancer [15]. However, to the best of our knowledge, the relationship between gallbladder disease and gastric polyps has not been defined yet. Herein, we examine the relationship between gallbladder disease and gastrointestinal polyps in China. If this study can prove an association between gallbladder disease and gastrointestinal polyps, it would pave the way for the use of endoscopic screening in patients with gallbladder diseases.

## Methods

### Study design and participants

This was a retrospective cross-sectional study involving all eligible patients who underwent a general check-up, which included gastroscopy, colonoscopy, and abdominal sonography between January 2015 and July 2020 at Changzhou No. 2 People’s Hospital Affiliated to Nanjing Medical University, China. The patients were divided into four groups based on the endoscopic findings: polyp-free (no gastric or colorectal polyp), only gastric polyp, only colorectal polyp, and gastric plus colorectal polyp (both gastric and colorectal polyp). The inclusion criteria were as follows: more than 18 years old; no history of inflammatory bowel disease, intestinal tuberculosis, familial colorectal polyposis syndrome, gastrointestinal polyps, gastrointestinal tumors, gastrointestinal surgery, or cholecystectomy; adequate bowel preparation; and no missing data. The study protocol was approved by the Ethics Committee of Changzhou No. 2 People’s Hospital and complied with the Declaration of Helsinki. The need for informed consent was waived by the Ethics Committee of Changzhou No. 2 People’s Hospital owing to its retrospective study design.

### Endoscopy

Screening gastroscopy (GIF-Q260, Olympus, Tokyo, Japan) and colonoscopy (CF-H260AI, Olympus, Tokyo, Japan) using a conventional white light endoscope were performed at Changzhou No. 2 People’s Hospital Affiliated to Nanjing Medical University. The endoscopy procedures were performed by experienced endoscopy examiners. To prepare the bowel for colonoscopy, subjects were required to drink 4 L Polyethylene glycol until clear rectal fluid was evacuated. The procedures were performed under conscious sedation with intravenous propofol. The polyp size was determined by comparison to the size of an opened endoscopic forceps and the largest one was measured when there were two or more gastrointestinal polyps. The size, location, and number of gastric and colonic lesions were recorded. All detected gastrointestinal polyps were biopsied or polypectomized for histopathologic examination based on the World Health Organization classification. The colorectal polyps were recorded as being in the right colon (proximal to splenic flexure) or left colon (splenic flexure and distal to splenic flexure).

### Abdominal ultrasound

Experienced radiologists specializing in abdominal ultrasonography performed abdominal ultrasound examinations (the Philips EPIQ ultrasound system). The participants were examined in the fasting state in the morning. We defined gallstones as ultrasound-documented gallstones depending on whether the strong intraluminal echoes were gravity-dependent or whether they attenuated ultrasound transmission (acoustic shadowing) [16]. Gallbladder polyps were defined as ultrasound-documented gallbladder polyps based on the presence of a hyperechoic immobile elevation of the gallbladder wall protruding into the lumen without acoustic shadowing [5]. A diagnosis of hepatic steatosis was made by using four known criteria: hepatorenal echogenic contrast, liver brightness, deep attenuation, and vascular blurring [17].

### Definitions and exposure measurements

Data on the age, sex, body mass index (BMI), family history of CRC, history of hypertension (systolic pressure ≥ 140 mmHg and/or diastolic ≥ 90 mmHg), history of diabetes, smoking (at least 1 cigarette per day for more than 1 year), alcohol consumption (at least 70 g per week), hepatic steatosis, gallbladder polyps, gallstones (detected by abdominal ultrasonography), glycosylated hemoglobin, fasting plasma glucose, and dyslipidemia were collected. Blood glucose and lipid profiles were measured in the fasting state.

### Statistical analysis

All statistical analyses were performed using SPSS version 23 (IBM, Armonk, NY, USA). We presented symmetrically distributed continuous variables as means with standard deviations and categorical variables as frequencies and percentages. We used the Pearson’s chi-squared test or Fisher’s exact test for the analysis of categorical variables and the Student’s *t*-test for continuous variables to compare the baseline characteristics of the participants in each group. Logistic regression analysis was performed to determine the odds ratio (OR) and 95% confidence intervals (CI). Univariate analysis was applied for potentially relevant variables that differed between each group. Multivariable analysis was performed for those significant (*P* < .05) in the univariate analysis. We conducted subgroup analysis based on the size, number of gallbladder polyps, location, pathology of colorectal polyps, age and gender. All two-tailed tests with *P*-values of <0.05 were considered meaningful.

## Results

The four groups contained 848 men and 814 women: polyp-free group (423 men and 535 women), colorectal polyp group (342 men and 162 women), gastric polyp group (31 men and 78 women), and gastric plus colorectal polyp group (52 men and 39 women). Gallbladder polyps and gallstones had a prevalence of 10.5% and 9.6%, respectively. Baseline characteristics were compared and the results are shown in Table 1. There were significant differences in the age, sex, BMI, gallbladder polyps, high-density lipoprotein cholesterol (HDL-C), hepatic steatosis, history of smoking, alcohol consumption, and diabetes between the colorectal polyp group and the polyp-free group. Significant differences were also noted in the total cholesterol (TC), glycosylated hemoglobin levels, and sex between the polyp-free group and the gastric polyp group, as well as in the age, BMI, gallbladder polyps, hepatic steatosis, HDL-C, and history of hypertension between the polyp-free group and the gastric plus colorectal polyp group. In contrast, no significant difference was observed in the prevalence of gallstones between colorectal polyp group (10.7%, 54/504) and polyp-free group (9%, 87/958, P = .315), gastric polyp group (8.3%, 9/109) and polyp-free group (9%, 87/958, P = .974), and gastric plus colorectal polyp group (13.5%, 10/91) and polyp-free group (9%, 87/958, P = .598).

**Table 1:** Comparison of baseline characteristics among individuals with and without gastric or colorectal polyps

Variables	Polyp-free	Colorectal polyps	*P*-value^a^	Gastric polyps	*P*-value^b^	Gastric plus colorectal polyps	*P*-value^c^
Age (years)	53.94±11.14	57.20±9.50	<.001	54.26±11.88	.786	57.52±10.23	.003
Sex (%male)	423 (44.1%)	342 (67.8%)	<.001	31 (28.4%)	.002	52 (57.1%)	.017
BMI (kg/m^2^)	23.54±2.94	24.12±2.79	<.001	23.75±2.65	.482	24.21±2.83	.038
Family history of cancer (%)	9 (0.9%)	3 (0.5%)	.761	2 (1.8%)	.312	1 (1%)	.598
Smoking (%)	121 (12.6%)	110 (21.8%)	<.001	7 (6.4%)	.059	10 (10.9%)	.651
Alcohol consumption (%)	77 (8%)	64 (12.6%)	.004	4 (3.6%)	.126	10 (10.9%)	.329
History of hypertension (%)	294 (30.6%)	207 (41%)	<.001	34 (31.1%)	.914	45 (49.4%)	<.001
History of diabetes (%)	144 (15%)	103 (20.4%)	.014	11 (10%)	.166	16 (17.5%)	.518
Presence of GB polyps (%)	77 (8%)	67 (13.2%)	.001	14 (12.8%)	.089	17 (18.6%)	.001
Presence of GB stone (%)	87 (9%)	54 (10.7%)	.315	9 (8.3%)	.974	10 (13.5%)	.598
Presence of hepatic steatosis (%)	328 (34.2%)	201 (39.8%)	.033	33 (30.2%)	.407	41 (45%)	.039
Fasting plasma glucose (mmol/L)	5.50±1.72	5.64±1.63	.154	5.36±1.34	.387	5.78±1.85	.146
Triglycerides (mmol/L)	1.70±1.36	1.79±1.58	.251	1.55±1.03	.252	2.00±2.53	.261
Total cholesterol (mmol/L)	4.84±1.20	4.73±1.07	.080	4.59±1.05	.041	4.94±1.17	.415
HDL-C (mmol/L)	1.34±0.38	1.29±0.39	.008	1.33±0.33	.687	1.27±0.32	.031
LDL-C (mmol/L)	2.63±0.88	2.65±0.77	.742	2.49±0.78	.112	2.72±0.85	.337
Glycosylated hemoglobin (mmol/L)	6.05±1.13	6.14±1.04	.144	5.83±0.73	.006	6.39±1.62	.053

*BMI* Body mass index, *GB* Gallbladder, *HDL-C* High-density lipoprotein cholesterol, *LDL-C* Low-density lipoprotein cholesterol

^a^ Polyp-free group VS Colorectal polyp group

^b^ Polyp-free group VS Gastric polyp group

^c^ Polyp-free group VS Gastric plus colorectal polyp group

Table 2 presents the OR and 95% CI of the risk factors for gastrointestinal polyps. After conducting multivariable adjustments, it was found that patients with gallbladder polyps had greater odds of having colorectal polyps (adjusted OR = 1.77, 95% CI: 1.23–2.54, *P* = .002) than those without. Similarly, those with gallbladder polyps had greater odds of having gastric plus colorectal polyps (adjusted OR = 2.94, 95% CI: 1.62–5.32, *P* < .001) than those without.

**Table 2:** Multivariate analysis of risk factors for gastrointestinal polyps

Variables	CP vs polyp-free		GP vs polyp-free		GP plus CP vs polyp-free	
	Multivariable analysis		Multivariable analysis		Multivariable analysis	
	OR (95%Cl)^a^	*P*-value	OR (95%Cl) ^b^	*P*-value	OR (95%Cl) ^c^	*P*-value
Age, year	1.03 (1.02,1.04)	<.001			1.03 (1.01,1.05)	.018
Male vs female	2.41 (1.87,3.12)	<.001	0.50 (0.32,0.77)	.002	1.51 (0.95,2.38)	.079
History of hypertension	1.09 (0.85,1.41)	.497			1.68 (1.05,2.71)	.032
Presence of GB polyps	1.77 (1.23,2.54)	.002			2.94 (1.62,5.32)	<.001
TC > 5.7 mmol/L			0.53 (0.28,0.98)	.043		

*CP* Colorectal polyp, *GP* Gastric polyp, *BMI* Body mass index, *GB* Gallbladder, *HDL-C* High-density lipoprotein cholesterol, *TC* Total cholesterol

^a^Adjusted for BMI, smoking, alcohol consumption, diabetes, hepatic steatosis and HDL-C.

^b^Adjusted for glycosylated hemoglobin.

^c^Adjusted for BMI and HDL-C.

The risk of gastrointestinal polyps according to the size and number of gallbladder polyps are shown in Table 3. Subgroup analysis revealed that individuals with gallbladder polyps ≥ 0.5 cm (adjusted OR = 2.21, 95% CI: 1.35–3.64, *P* = .002) or multiple gallbladder polyps (adjusted OR = 2.33, 95% CI: 1.33–4.07, *P* = .003) had a higher prevalence of colorectal polyps. Moreover, subgroup analysis also revealed that individuals with multiple gallbladder polyps (adjusted OR = 3.95, 95% CI: 1.72–9.11, *P* = .001) or gallbladder polyps of any size (gallbladder polyps < 0.5 cm: adjusted OR = 2.56, 95% CI: 1.19–5.54, *P* = .017; gallbladder polyps ≥ 0.5 cm: adjusted OR = 3.16, 95% CI: 1.39–7.17, *P* = .006) had a higher prevalence of gastric plus colorectal polyps. In contrast, there was no correlation between the number and size of gallbladder polyps and gastric polyps.

**Table 3:** Risk of gastrointestinal polyps according to the size and number of gallbladder polyps

Variables	CP vs polyp-free				GP plus CP vs polyp-free			
	OR (95%Cl)	*P*-value	Adjusted OR (95%Cl)^a^	*P*-value	OR (95%Cl)	*P*-value	Adjusted OR (95%Cl) ^b^	*P*-value
GB polyps								
Size								
No GB polyp	1		1		1		1	
<0.5 cm	1.52 (0.94,2.46)	.085	1.28 (0.78,2.11)	.327	2.61 (1.22,5.59)	.013	2.56 (1.19,5.54)	.017
≥0.5 cm	2.02 (1.25,3.24)	.004	2.21 (1.35,3.64)	.002	2.64 (1.19,5.90)	.017	3.16 (1.39,7.17)	.006
Number								
No GB polyp	1		1		1		1	
Single	1.56 (1.01,2.43)	.046	1.37 (0.87,2.16)	.176	2.19 (1.03,4.63)	.041	2.13 (1.00,4.52)	.050
Multiple (≥2)	2.09 (1.23,3.55)	.007	2.33 (1.33,4.07)	.003	3.40 (1.50,7.73)	.003	3.95 (1.72,9.11)	.001

*CP* Colorectal polyp, *GP* Gastric polyp, *GB* Gallbladder

^a^ Adjusted for age and sex

^b^Adjusted for age and hypertension

The risk factors for colonic polyps at different locations were reported in supplement table 1. There is a borderline positive association between gallbladder polyps and right-colon polyps (adjusted OR = 1.77, 95% CI: 0.97–3.23, P = .064). After conducting multivariable adjustments, it was observed that patients with gallbladder polyps had greater odds of having left-colon polyps (adjusted OR = 1.90, 95% CI: 1.25–2.88, *P* = .003) than those without. The risk factors for colorectal polyps with different pathologies were reported in supplement table 2. No correlation was found between gallbladder polyps and colorectal non-adenoma (hyperplastic or inflammatory polyps) (OR = 1.55, 95% CI: 0.91–2.65, *P* = .105). Again, after conducting multivariable adjustments, we noted that patients with gallbladder polyps had a higher prevalence of colorectal adenoma (adjusted OR = 1.78, 95% CI: 1.19–2.66, *P* = .005) than those without.

Subgroup analysis showed that patients with gallbladder polyps ≥ 0.5 cm (adjusted OR = 2.28, 95% CI: 1.28–4.05, *P* = .005) and multiple gallbladder polyps (adjusted OR = 2.37, 95% CI: 1.23–4.56, *P* = .010) had a higher prevalence of left-colon polyps (Table 4). Subgroup analysis showed also that patients with gallbladder polyps ≥ 0.5 cm (adjusted OR = 1.94, 95% CI: 1.10–3.42, *P* = .023) and multiple gallbladder polyps (adjusted OR = 2.10, 95% CI: 1.11–3.98, *P* = .023) had a higher prevalence of colorectal adenoma than those without (Table 4).

**Table 4:** Risk of colorectal polyps with different locations and pathology according to the size and number of gallbladder polyps

Variables	Left-colon polyp vs polyp-free				Right-colon polyp vs polyp-free				Colorectal adenoma vs polyp-free			
	OR (95%Cl)	*P*-value	Adjusted OR (95%Cl)^a^	*P*-value	OR (95%Cl)	*P*-value	Adjusted OR (95%Cl)^a^	*P*-value	OR (95%Cl)	*P*-value	Adjusted OR (95%Cl)^a^	*P*-value
GB polyps												
Size												
No GB polyp	1		1		1		1		1		1	
<0.5 cm	1.75 (1.01,3.04)	.048	1.53 (0.87,2.70)	.140	1.64 (0.71,3.75)	.246	1.44 (0.62,3.33)	.396	1.88 (1.13,3.13)	.015	1.58 (0.93,2.69)	.089
≥0.5 cm	2.09 (1.20,3.64)	.009	2.28 (1.28,4.05)	.005	2.13 (0.96,4.72)	.063	2.10 (0.93,4.72)	.073	1.81 (1.05,3.13)	.033	1.94 (1.10,3.42)	.023
Number												
No GB polyp	1		1		1		1		1		1	
Single	1.83 (1.11,3.02)	.019	1.61 (0.96,2.70)	.069	1.76 (0.84,3.70)	.136	1.53 (0.72,3.25)	.266	1.82 (1.13,2.92)	.013	1.56 (0.96,2.55)	.075
Multiple (≥2)	2.05 (1.09,3.84)	.026	2.37 (1.23,4.56)	.010	2.05 (0.83,5.09)	.121	2.13 (0.85,5.38)	.108	1.91 (1.04,3.50)	.037	2.10 (1.11,3.98)	.023

*GB* gallbladder

^a^Adjusted for age and sex

We also compared the association between gastrointestinal polyps and gallbladder polyps based on sex and age (Table 5). Women with gallbladder polyps had a higher likelihood of developing both colorectal polyps (OR = 2.13, 95% CI: 1.20–3.77, *P* = .010) and gastric plus colorectal polyps (OR = 3.69, 95% CI: 1.58–8.62, *P* = .003). In patients aged ≥ 50 years with gallbladder polyps had a higher likelihood of developing both colorectal polyps (OR = 1.58, 95% CI: 1.04–2.38, *P* = .031), and gastric plus colorectal polyps (OR = 2.58, 95% CI: 1.32–5.03, *P* = .005).

**Table 5:** Risk of gastrointestinal polyps with and without gallbladder polyps according to sex and age

Variables	CP vs polyp-free		GP vs polyp-free		GP plus CP vs polyp-free	
	OR (95%Cl)	*P*-value	OR (95%Cl)	*P*-value	OR (95%Cl)	*P*-value
GB polyp						
Sex						
Male	1.41 (0.90,2.20)	.130	1.75 (0.64,4.78)	.280	1.90 (0.87,4.17)	.110
Female	2.13 (1.20,3.77)	.010	1.86 (0.86,4.04)	.115	3.69 (1.58,8.62)	.003
Age						
<50 years	2.48 (1.30,4.73)	.006	1.26 (0.42,3.82)	.678	2.75 (0.86,8.78)	.087
≥50 years	1.58 (1.04,2.38)	.031	1.95 (0.94,4.05)	.073	2.58 (1.32,5.03)	.005

*CP* Colorectal polyp, *GP* Gastric polyp, *GB* Gallbladder

## Discussion

To the best of our knowledge, this is the first study that investigates the association between gallbladder diseases and gastrointestinal polyps. This retrospective study found gallbladder polyps to be positively associated with gastrointestinal polyps, especially with multiple or larger gallbladder polyps.

Our study showed that gallbladder polyps are positively related to colorectal adenoma. However, the underlying mechanism for the association could not be established. Both gallbladder polyps and colorectal adenoma have some common risk factors such as age, male, obesity, and metabolic syndrome [6, 7]. An analysis of 21771 individuals by Segawa et al. [18] showed that obesity can lead to gallbladder polyps. Kim et al. [7] analyzed 1316 individuals and suggested obesity was an independent risk factor for developing colorectal adenoma. Lim et al. showed that patients with metabolic syndrome had a 2.35-fold higher risk of developing gallbladder polyps than those without [6]. Milano et al. [19] conducted a multicenter cross-sectional study on 5707 patients and showed that metabolic syndrome increased the risk of colorectal adenoma by 1.76-fold. Therefore, the association between gallbladder polyps and colorectal adenoma may be due to the same risk factors and both diseases may develop through a similar pathway. We also speculate that gallbladder polyps and colorectal polyps could be related to bile acids. There is evidence that higher levels of secondary bile acids are associated with gallbladder polyps [20]. Imray et al. [21] reported a higher concentration of fecal bile acids, lithocholic acid, and total secondary bile acids in patients with adenomatous polyps than in normal individuals. In addition, it has been suggested that cholesterol 7α-hydroxylase, the rate-limiting enzyme in the conversion of cholesterol to bile acids, is the determining risk factor for developing colorectal adenoma [22]. Studies have shown that patients with colorectal polyps or colorectal adenoma have significantly high levels of acteroidetes [23], which also increases the risk of CRC development [24]. It has been shown that the crosstalk between bile acids and Bacteroidetes can catalyze the development of adenoma and eventual CRC [23, 25]. Our subgroup analysis showed that gallbladder polyps are positively related to left-colon polyps. This observation can be explained by the relatively longer stasis of feces in the left colon, resulting in prolonged exposure of epithelial cells to bile acids. Consequently, bacterial modulation increases the production of more cytotoxic secondary bile acids in the left colon than that produced in the right colon.

In this study, gallbladder polyps were found to be significantly associated with gastric plus colorectal polyps. However, the mechanism underlying the relationship between gallbladder polyps and gastric plus colorectal polyps could not be determined. This association may result from exposure to the same risk factors. Lim et al. reported age as a risk factor for gallbladder polyps [6]. Other studies have also shown that age is a risk factor for gastric plus colorectal polyps [26, 27]. Metabolic syndrome is associated with an increased risk of gallbladder polyps and gastrointestinal polyps [6, 19, 28]. The possible association between these two conditions should be further investigated in larger studies.

We found that in female patients, gallbladder polyps were associated with gastrointestinal polyps. However, Liu et al. [10] showed that gallbladder polyps increase the risk of colorectal adenomas in men, but not in women. This inconsistent result may be related to postmenopausal hormone levels in women. After comparing the baseline data, the average age of patients in our study was >55 years. A cross-section study [29] showed that the overall median age of Chinese women at natural menopause was 50 years. Menopause is accompanied by a significant decline in sex hormone levels. A meta-analysis of 31 studies showed that menopause adversely affected nearly all components of metabolic syndrome [30]. Metabolic syndrome is also associated with gallbladder polyps and gastrointestinal polyps [6, 19, 28]. In addition, this difference between the association of gallbladder polyps and gastrointestinal polyps may be caused by differences in the genetic backgrounds of the study population. We conducted our study in Jiangsu, China, while Liu et al. conducted theirs in Taiwan, China. The populations in these two regions have different hereditary backgrounds. For example, a case-control study [31] confirmed that the DNA repair gene (*XRCC1 399Arg/Gln, 399Gln/Gln genotype*) increases the susceptibility of the population of Jiangsu, China toward CRC. In contrast, no significant correlation between this polymorphism and CRC susceptibility has been reported in Taiwan, China [32].

No correlation could be found between gallstones and colorectal polyps. The difference in the correlation between gallstones and colorectal polyps between our study and the other two studies [10, 12] may be due to different age distributions and genetic backgrounds. In our study, the average age of patients with gastrointestinal polyps was >55 years, whereas it was ≤55 years in the other two studies. In addition, our study was conducted on the mainland, while the other two studies were conducted outside of it. Differences in the genetic backgrounds are possible reasons for the difference in the results.

Our study has some limitations as well. First, it is a single-center retrospective study. Therefore, the effects of a potential selection bias cannot be completely excluded. In addition, the number of patients in some groups, especially that in the gastric polyp group (only 14 of 109 patients with gastric polyps had gallbladder polyps) and gastric plus colorectal polyp group (only 17 of 91 patients with gastric plus colorectal polyps had gallbladder polyps), may not be sufficient and the statistical power is relatively low, which precluded any meaningful subgroup analyses. Second, *Helicobacter pylori* infection is a well-known risk factor for gastric hyperplastic polyps, and gastric cancer. Despite this, we did not include *H. pylori* infection while analyzing the risk factors for gastric polyps because of incomplete data due to the retrospective nature of the study. Third, we could not confirm the histopathology of gallbladder polyps because we obtained our data from patients who underwent ultrasound examinations only as part of their routine health check-ups. Finally, although we believe that the hormone levels of menopausal women impacts the growth of colorectal adenoma and CRC, we did not monitor them in these women. Nonetheless, this is the first study to investigate the association between gallbladder diseases and gastrointestinal polyps in China. Further large-scale, multicenter prospective studies are warranted to verify our results.

## Conclusion

The presence of gallbladder polyps is significantly associated with colorectal polyps and gastric plus colorectal polyps. Therefore, screening endoscopy should be considered for patients with gallbladder polyps, especially for those with gallbladder polyps ≥0.5 cm or multiple gallbladder polyps and for female patients.

## Supplementary Information

**Additional file 1.**

## Data Availability

All data used to support the findings of this study are included in this article.

## Abbreviations

CRC: Colorectal cancer
OR: Odds ratio
Cl: Confidence interval
BMI: Body mass index
HDL-C: High-density lipoprotein cholesterol
TC: Total cholesterol
DNA: Deoxyribonucleic acid
