# Supplementary material for: Association of gallbladder diseases with risk of gastrointestinal polyps
Source: BMC Gastroenterol. 2022 Nov 21;22:476. doi: 10.1186/s12876-022-02566-6 (PMC9677685; doi:10.1186/s12876-022-02566-6)
Supplement: Supplementary file 1 — Additional file 1. [file 12876_2022_2566_MOESM1_ESM.docx]

Supplement Table 1. Risk factors for colon polyps in different locations

| Variables | Left-colon polyp vs polyp-free | | | | Right-colon polyp vs polyp-free | | | |
| --- | --- | --- | --- | --- | --- | --- | --- | --- |
|  | OR (95%Cl) | *P*-value | Adjusted OR  (95%Cl) | *P*-value | OR (95%Cl) | *P*-value | Adjusted OR  (95%Cl) | *P*-value |
| Age, year | 1.02 (1.01,1.04) | <.001 | 1.03 (1.01,1.04) | <.001 | 1.03 (1.01,1.05) | .002 | 1.03 (1.01,1.05) | .005 |
| Male vs female | 2.40 (1.83,3.16) | <.001 | 2.26 (1.67,3.05) | <.001 | 1.81 (1.21,2.72) | .004 | 1.79 (1.18,2.71) | .006 |
| BMI ≥ 24 kg/m² | 1.21 (0.93,1.58) | .154 |  |  | 1.44 (0.97,2.16) | .072 |  |  |
| Fasting plasma glucose ≥ 7 mmol/L | 1.21 (0.79,1.87) | .383 |  |  | 1.51 (0.82,2.76) | .183 |  |  |
| Total cholesterol > 5.7 mmol/L | 0.98 (0.69,1.37) | .891 |  |  | 1.01 (0.61,1.69) | .957 |  |  |
| Triglyceride > 1.7 mmol/L | 1.16 (0.88,1.53) | .303 |  |  | 1.13 (0.74,1.72) | .580 |  |  |
| HDL-C < 1.09 mmol/L | 1.17 (0.88,1.57) | .277 |  |  | 1.43 (0.93,2.18) | .100 |  |  |
| LDL-C > 3.61 mmol/L | 1.06 (0.69,1.64) | .793 |  |  | 0.87 (0.44,1.71) | .678 |  |  |
| History of hypertension | 1.42 (1.08,1.87) | .012 | 1.08 (0.80,1.46) | .615 | 1.64 (1.09,2.46) | .017 | 1.29 (0.83,1.99) | .254 |
| History of diabetes | 1.31 (0.93,1.85) | .125 |  |  | 1.38 (0.83,2.30) | .214 |  |  |
| Smoking | 1.91 (1.36,2.68) | <.001 | 1.40 (0.91,2.17) | .130 | 1.22 (0.69,2.14) | .497 |  |  |
| Alcohol consumption | 1.59 (1.04,2.43) | .032 | 0.83 (0.49,1.40) | .475 | 1.31 (0.67,2.55) | .425 |  |  |
| Presence of GB stones | 1.17 (0.75,1.81) | .485 |  |  | 1.51 (0.82,2.76) | .183 |  |  |
| Presence of GB polyps | 1.91 (1.27,2.86) | .002 | 1.90 (1.25,2.88) | .003 | 1.87 (1.03,3.38) | .039 | 1.77 (0.97,3.23) | .064 |
| Presence of hepatic steatosis | 1.21 (0.92,1.59) | .168 |  |  | 1.29 (0.86,1.94) | .222 |  |  |

CP, colorectal polyp; GP, gastric polyp; BMI, body mass index; GB, gallbladder; HDL-C, high-density lipoprotein cholesterol; TC, Total cholesterol.

Supplement Table 2. Risk factors for colorectal polyps with different pathology

| Variables | Colorectal adenoma vs polyp-free | | | | Colorectal non-adenoma vs polyp-free | | | |
| --- | --- | --- | --- | --- | --- | --- | --- | --- |
|  | OR (95%Cl) | *P*-value | Adjusted OR  (95%Cl) | *P*-value | OR (95%Cl) | *P*-value | Adjusted OR  (95%Cl) | *P*-value |
| Age, year | 1.03 (1.02,1.05) | <.001 | 1.03 (1.02,1.05) | <.001 | 1.02 (1.01,1.04) | .008 | 1.02 (1.01,1.04) | .008 |
| Male vs female | 2.70 (2.08,3.50) | <.001 | 2.45 (1.84,3.28) | <.001 | 2.60 (1.83,3.71) | <.001 | 2.42 (1.63,3.58) | <.001 |
| BMI ≥ 24 kg/m² | 1.47 (1.15,1.89) | .002 | 1.17 (0.90,1.53) | .244 | 1.45 (1.04,2.04) | .029 | 1.08 (0.74,1.56) | .697 |
| Fasting plasma glucose ≥ 7 mmol/L | 1.20 (0.80,1.81) | .373 |  |  | 1.44 (0.86,2.42) | .167 |  |  |
| Total cholesterol > 5.7 mmol/L | 0.84 (0.60,1.17) | .292 |  |  | 0.94 (0.61,1.46) | .794 |  |  |
| Triglyceride > 1.7 mmol/L |  |  |  |  | 1.26 (0.89,1.78) | .199 |  |  |
| HDL-C < 1.09 mmol/L | 1.21 (0.94,1.57) | .147 |  |  | 1.47 (1.03,2.09) | .034 | 1.07 (0.73,1.58) | .725 |
| LDL-C > 3.61 mmol/L | 1.32 (1.01,1.72) | .044 | 0.94 (0.61,1.44) | .774 | 0.94 (0.54,1.64) | .824 |  |  |
| History of hypertension | 1.64 (1.27,2.11) | <.001 | 1.12 (0.85,1.49) | .421 | 1.44 (1.02,2.04) | .038 | 0.98 (0.67,1.44) | .919 |
| History of diabetes | 1.21 (0.87,1.69) | .247 |  |  | 2.03 (1.37,3.01) | <.001 | 1.55 (1.01,2.38) | .045 |
| Smoking | 1.95 (1.42,2.69) | <.001 | 1.21 (0.80,1.83) | .363 | 1.88 (1.23,2.88) | .004 | 1.09 (0.69,1.75) | .706 |
| Alcohol consumption | 1.94 (1.33,2.84) | .001 | 1.07 (0.67,1.74) | .770 | 1.11 (0.61,2.01) | .743 |  |  |
| Presence of GB stones | 1.20 (0.83,1.85) | .297 |  |  | 1.12 (0.64,1.96) | .692 |  |  |
| Presence of GB polyps | 1.85 (1.26,2.71) | .002 | 1.78 (1.19,2.66) | .005 | 1.55 (0.91,2.65) | .105 |  |  |
| Presence of hepatic steatosis | 1.18 (0.91,1.52) | .213 |  |  | 1.51 (1.08,2.12) | .018 | 1.29 (0.89,1.87) | .182 |

CP, colorectal polyp; GP, gastric polyp; BMI, body mass index; GB, gallbladder; HDL-C, high-density lipoprotein cholesterol; TC, Total cholesterol.
